# Thioredoxin-1 Is a Target to Attenuate Alzheimer-Like Pathology in Diabetic Encephalopathy by Alleviating Endoplasmic Reticulum Stress and Oxidative Stress

**DOI:** 10.3389/fphys.2021.651105

**Published:** 2021-05-17

**Authors:** Yu Guo, Chenghong Zhang, Chunyang Wang, Yufei Huang, Jingyun Liu, Haiying Chu, Xiang Ren, Li Kong, Haiying Ma

**Affiliations:** Department of Histology and Embryology, College of Basic Medical Sciences, Dalian Medical University, Dalian, China

**Keywords:** diabetic encephalopathy, Alzheimer’s disease, thioredoxin-1, endoplasmic reticulum stress, oxidative stress

## Abstract

Varying degrees of central nervous system neuropathy induced by diabetes mellitus (DM) contribute to a cognitive disorder known as diabetic encephalopathy (DE), which is also one of the independent risk factors for Alzheimer’s disease (AD). Endoplasmic reticulum stress (ERS) plays a critical role in the occurrence and development of DE and AD. However, its molecular mechanism remains largely unknown. This study aims to investigate whether thioredoxin-1 (Trx-1) could alleviate DE and AD through ERS, oxidative stress (OS) and apoptosis signaling pathways. Mice were randomly divided into a wild-type group (WT-NC), a streptozotocin (STZ)-treated DM group (WT-DM), a Trx-1-TG group (TG-NC) and a Trx-1-TG DM group (TG-DM). Diabetic animals showed an increase in the time spent in the target quadrant and the number of platform crossings as well as AD-like behavior in the water maze experiment. The immunocontent of the AD-related protein Tau and the levels of cell apoptosis, β-amyloid (Aβ) plaque formation and neuronal degeneration in the hippocampus of the diabetic group were increased. Some key factors associated with ERS, such as protein disulfide isomerase (PDI), glucose-regulated protein 78 (GRP78), inositol-requiring enzyme 1α (IRE1α), tumor necrosis factor receptor-associated factor 2 (TRAF2), apoptosis signal-regulating kinase-1 (ASK1), c-Jun N-terminal kinase (JNK), protein kinase RNA (PKR)-like ER kinase (PERK), and C/EBP homologous protein (CHOP), were upregulated, and other factors related to anti-oxidant stress, such as nuclear factor erythroid 2-related factor (Nrf2), were downregulated in the DM group. Moreover, DM caused an increase in the immunocontents of caspase-3 and caspase-12. However, these changes were reversed in the Trx-1-tg DM group. Therefore, we conclude that Trx-1 might be a key factor in alleviating DE and AD by regulating ERS and oxidative stress response, thus preventing apoptosis.

## Introduction

Many studies have shown that both type 1 and type 2 diabetes can cause varying degrees of central nervous system dysfunction and cognitive decline, which is called diabetic encephalopathy (DE) (Stranahan, 2015). The molecular mechanism of the cognitive dysfunction in DE has been widely studied, but it is still not clear. A large number of studies have shown that endoplasmic reticulum stress (ERS) and oxidative stress (OS) are involved in the pathogenesis of DE (Biessels et al., 2002; Díaz-Gerevini et al., 2014; Wang et al., 2016). Lupachyk et al. (2013) found CHOP deficient mice displayed improved sciatic nerve oxidative-nitrative stress and attenuated peripheral neuropathy as compared with their wild-type (WT) littermates in the setting of diabetes, suggesting dysregulated UPR induced by prolonged ER stress is implicated in the development of diabetic peripheral neuropathy. While another research shown that the up-regulation of CHOP in hippocampal neurons of diabetic mice may promote neuronal apoptosis and account for the damaged learning and memory ability of diabetic mice (Zhao et al., 2015). Dabidi et al. found that OS affects brain tissue with the progress of the disease course, the damage of brain tissue gradually accumulation, degeneration and even necrosis of nerve cells, and then central nervous system DE occurs as a result of chronic, irreversible damage to the system (Roshan et al., 2013).

A large number of epidemiological studies have shown that diabetes significantly increases the risk of acquiring Alzheimer’s disease (AD); pathological changes similar to AD, such as Aβ deposits, neurofibrillary tangles, and damaged or lost neuronal synapses, are also found in patients with DE (Selkoe, 2001; Zargar et al., 2014). Other studies have shown that diabetes can promote the abnormal modification of Tau protein and that ERS and OS are also involved in the pathogenesis of AD: an upregulation of PDI during ER stress associated with multiple neurodegenerative diseases including AD represents an adaptive response to protect neuronal cells, their results here indicate that human PDI, a key component of the protein quality control system in the endoplasmic reticulum (ER) (Han et al., 2009; Xu et al., 2013), can directly interact with human Tau on the ER, and could counteract the toxicity caused by abnormal Tau aggregation in the ER by binding to Tau protein and inhibiting Tau fibrillization (Clodfelder-Miller et al., 2006; Asano et al., 2007; Aubert et al., 2015). In addition, most studies indicate that Tau is involved in the neurodegeneration associated with oxidative stress in AD: in a Drosophila model of human tauopathy (Tau R406W), a reduction in the gene dosage of thioredoxin reductase or mitochondrial superoxide dismutase 2 (SOD2) promotes Tau-induced neurodegenerative histological abnormalities and neuronal apoptosis. On the contrary, overexpression of these antioxidant enzymes or treatment with vitamin E decreases the Tau-induced neuronal death (Stamer et al., 2002). These findings suggest that DE and AD may have the same mechanisms.

High glucose levels in the body disrupt the internal balance of the ER, resulting in the accumulation of unfolded or misfolded proteins, which leads to ERS (Jin et al., 2016; Wu et al., 2016), and the main response to ERS is the activation of the unfolded protein response (UPR) (Dan et al., 2017). At this point, most of the UPR signaling pathways are activated, including the following: (1) after inositol-requiring enzyme 1α (IRE1α) is activated, tumor necrosis factor receptor-associated factor 2 (TRAF2) forms a complex with apoptosis signal-regulating kinase-1 (ASK1), which activates c-Jun N-terminal kinase (JNK) and subsequently caspase-12 on the ER membrane to induce apoptosis; (2) activating transcription factor 6 (ATF6) is released from the ER membrane, which leads to its nuclear translocation and upregulation of ER stress response genes; (3) glucose-regulated protein 78 (GRP78) activation induces the protein kinase RNA (PKR)-like ER kinase (PERK) eukaryotic initiation factor 2α (Eif2α) pathway to activate the expression of C/EBP homologous protein (CHOP) to initiate apoptosis (Zahraa et al., 2015; Almanza et al., 2018); additionally, the activation of PERK signaling can induce a conformational change in nuclear factor erythroid 2-related factor (Nrf2) that triggers the dissociation of the Kelch-like protein 1 (keap1)-Nrf2 complex and regulates cell oxidation (Zhu et al., 2015). In addition, it has been reported that Nrf2 is involved in increasing endogenous antioxidant levels and inhibiting apoptosis (Lim et al., 2014; Glory and Averill-Bates, 2016). It can be seen that there is a clear interaction between OS and ERS. OS exacerbates ERS, while ERS can also cause and exacerbate OS (Thummayot et al., 2016; Yang et al., 2016).

Thioredoxin-1 (Trx-1) is a small 12 kDa multifunctional protein having a redox-active disulfide/dithiol within its active site sequence, -Cys-Gly-Pro-Cys-, and operates together with NADPH and thioredoxin reductase (Holmgren, 1985). In the regulatory region of the Trx-1 gene, there are several promoter-specific transcription factor 1 binding motifs, an antioxidant-responsive element, and a putative cAMP-responsive element (CRE) (Bai et al., 2003). Trx-1 as a disulfide-reducing system low molecular weight protein with redox properties, and it plays an important role in regulating redox reactions in the human body (Matés and Sánchez-Jiménez, 2000; Bai et al., 2003). Trx-1, a major isoform of Trx that is ubiquitously expressed in many cell types and is found in various subcellular localizations (Nagarajan et al., 2016), has been reported to be a regulator of ERS (Bai et al., 2007). Some studies have also shown that Trx-1 can protect nerves by inhibiting the stress response of the ER (Luo et al., 2012; Zeng et al., 2014). During the pathogenic process of DM, long-term high glucose stimulation promotes the occurrence and development of ERS and OS, inhibits the activity of Trx-1, causes the dissociation of the Trx-1-ASK1 complex, activates the ASK1-mediated apoptotic pathway, and causes cell apoptosis (Zhu et al., 2018). As shown in several studies that: a decrease in neuronal Trx-1 in AD brains, Aβ neurotoxicity might be mediated by oxidation of Trx-1 and subsequent activation of the ASK1 cascade (Akterin et al., 2006). Deregulation of Trx-1 antioxidant systems could be important events in AD pathogenesis. Moreover, Trx-1 is also considered to be one of the antioxidant enzymes regulated by Nrf2 (Nakamura, 2004). Therefore, we reasoned that Trx-1/Nrf2 could play a key protective role in DE.

In this study, we verified the AD-like pathological changes and cognitive dysfunction of DE, including AD-related protein expression, neuronal apoptosis and neuronal degeneration, in the mouse hippocampus. More importantly, the effects of Trx-1 upregulation on the abovementioned factors and ERS, OS were studied. Our results suggest that Trx-1 can alleviate DE and AD by regulating the ERS and OS induced by DM.

## Materials and Methods

### Animal Experiments

Male C57/B6 wild-type (WT) and C57/B6 human-Trx-1 transgenic (TG) mice approximately 6∼8 weeks of age were used in the experiments. Mice were obtained from the SPF Animal Center of Dalian Medical University, housed in plastic cages and maintained on a 12 h light/dark cycle with free access to food and water. Mice were randomly selected from the wild-type and transgenic groups for the diabetic model groups. The mice were randomly divided into a wild-type group (WT-NC), a streptozotocin (STZ)-induced DM group (WT-DM), a Trx-1-tg group (TG-NC) and a Trx-1-tg DM group (TG-DM) (*n* = 6). The diabetic group was fasted for 12 h and then intraperitoneally injected with streptozotocin (STZ) solution (50 mg/kg) for five consecutive days. Then, they were fed a normal diet; after 72 h, their blood glucose was measured on three consecutive days, and fasting was carried out 8 h before each measurement. When the blood glucose was greater than 11.1 mmol/L, the diabetic model was considered to be successfully established. The normal control group was injected intraperitoneally with the same amount of citric acid-sodium citrate buffer, and all experimental materials were obtained at 12 weeks after the completion of the model. All procedures were performed in accordance with the Guide for the Care and Use of Laboratory Animals of the National Institutes of Health (NIH), and all protocols were approved by the Institutional Animal Care and Use Committee of Dalian Medical University.

### Morris Water Maze

The Morris water maze (MWM) test was used to evaluate spatial learning and memory. The MWM consists of a circular pool with a diameter of 120 cm and a depth of 45 cm. The tank was divided into four equal quadrants with the platform (10 cm × 10 cm) located in the center of the northeast quadrant. The platform was hidden 1 cm below the water surface, and a non-toxic white dye was used to make the water opaque. The testing room was illuminated with a constant intensity light source and kept quiet during the experiments, and different figures and objects were hung on the walls (○, □, △ and ×). All swimming trials were recorded with a video camera suspended from the ceiling and analyzed using EthoVision XT 9.0 software (Noldus, Netherlands).

Acquisition tests were performed with the mice for five consecutive days. Each mouse was placed into the water in all four quadrants on each test day. During the experiment, the time for the mice to find the platform in the water was recorded. This time is the escape latency period. The longest swimming time for the mice that could not find the platform was 60 s. If the platform was not found within 60 s, the experimenter guided the mouse to the platform and allowed it to stay on the platform for 10 s. The probe test was performed 24 h after the last acquisition trial. In the probe test, the platform was removed from the tank, and the mice were allowed to swim freely for 60 s. Then, the visual platform experiment was immediately performed to test the swimming speed of the mice.

### *In situ* TdT-Mediated dUTP Nick-End Labeling Assay

The TdT-Mediated dUTP Nick-End Labeling (TUNEL) assay was performed on mice hippocampal tissues following the manufacturer’s instructions (TransGen Biotech, China). First, deparaffinized tissue sections were washed with phosphate-buffered saline (PBS) for 5 min. Then, 100 μl of cell permeation solution (0.1% Triton X-100) was added dropwise to the sample area to be tested, the tissue was incubated at room temperature for 30 min, and the excess liquid was removed. The tissues were incubated with 50 μl of a well-mixed labeling solution and 2 μl of terminal deoxynucleotidyl transferase (TDT) at 37°C for 60 min in the dark to allow the tailing reaction to occur and then washed with PBS three times for 5 min each. Cell permeation buffer was added to the sample area to be tested three times for 5 min each. Finally, the TUNEL-stained slides were observed immediately upon completion of the assay. Fluorescent cells were quantified by ImageJ software.

### Immunohistochemistry

The sections were first dewaxed and hydrated, and then the sections were subjected to citrate antigen repair. An appropriate amount of blocking endogenous peroxidase was added dropwise to each section, and the sections were incubated for 15 min at room temperature and then washed with PBS 3 times for 3 min each; the sections were blocked with goat serum solution for 15 min at room temperature and subsequently incubated overnight at 4°C with a rabbit Aβ (Aβ_1__–__40_, Aβ_1__–__42_, 1:200, novusbio) antibody. The cells were washed with PBS three times for 3 min each and then treated with an appropriate amount of biotin-labeled sheep anti-rabbit/mouse IgG, incubated for 15 min at room temperature, and washed with PBS 3 times for 3 min each. An appropriate amount of streptozotocin-peroxidase was added, incubated for 15 min at room temperature, and washed with PBS three times for 3 min each. Diaminobenzidine (DAB) solution was applied to the sections for 10 s–5 min, and the sections were washed three times with PBS. Hematoxylin was used to stain the cell nuclei. Five random slides were selected from each group, and five randomly selected visual fields in the hippocampal region from each slide were observed. The mean optical density was quantified by ImageJ software.

### Western Blot

The samples were thawed, washed in ice-cold PBS and sonicated in KeyGen lysis assay buffer (KeyGen Biotech, China). The samples were then sonicated, incubated on ice for 30 min and centrifuged at 10,000 × *g* for 20 min at 4°C. The protein concentration in the supernatant was determined by a Pierce BCA Protein Assay Kit (Life Technologies, United States). Equal quantities of protein were separated by 10% [for the ASK-1 (1:1,000, Cell Signaling Technology, United States), p-ASK-1, IRE1α (Wanleibio, China), p-IRE1α, TRAF2 (1:1,000, Wanleibio), JNK (1:1,000, Wanleibio), p-JNK (1:1,000, Wanleibio), GRP78 (1:1,000, Wanleibio), CHOP (1:1,000, Wanleibio), Nrf2 (1:1,000, Wanleibio), p-PERK, PERK (1:1,000, Wanleibio), Tau (1:1,000, Cell Signaling Technology), and p-Tau (1:1,000, Cell Signaling Technology) antibodies] or 12% SDS–PAGE (for the cleaved caspase-12 (1:1,000, Wanleibio), cleaved caspase-3 (1:1,000, Wanleibio) antibodies) and transferred to a polyvinylidene difluoride membrane (Millipore Corp., United States). The membrane was soaked in 5% skim milk (in PBS, pH 7.2) or 5% bovine serum albumin (BSA) (in PBS, pH 7.2) overnight at 4°C and then incubated with primary antibodies (1:1000) followed by peroxidase-conjugated anti-mouse or anti-rabbit IgG antibody (1:10,000, KPL, Gaithersburg, United States). The epitope was visualized by an ECL Western blot detection kit (Millipore, United States) and imaged with a ChemiDoc^TM^ XRS and Image Lab^TM^ Software (Bio-Rad Laboratories, Inc. Hercules, United States).

### Fluoro-JadeC Staining

Slides containing sections of brain tissue were dewaxed in xylene two times, immersed in 100% ethanol two times for 5 min each, immersed in 70% alcohol for 2 min, and then rinsed with fresh ddH_2_O two times for 1 min each. Nine parts ddH_2_O was mixed with 1 part Solution B (potassium permanganate); the slides were added to this mixture and incubated for 10 min. Then, eight parts ddH_2_O was mixed with one part Solution C (Fluoro-Jade) (Merck Millipore, United States) and 1 part Solution D (DAPI) and placed in a Coplin jar in the dark or low light; the slides were incubated in this solution for 10 min. The slides were then rinsed in ddH_2_O three times for 1 min each and dried on a slide warmer at 50–60°C for at least 5 min. The dry slides were then cleared by brief immersion in xylene. Finally, the FJC slides were observed immediately upon completion of the assay, and the fluorescence intensity was quantified by ImageJ software.

### Real-Time Quantitative PCR

Total RNA was extracted using TRIzol reagent (Takara, China) and transcribed using a Reverse Transcription Kit (Applied Biosystems, United States) according to the manufacturer’s instructions. mRNA samples were mixed with primers and 2× TransStart Top Green qPCR SuperMix (Transgene) in a total volume of 20 μl. The thermal cycling conditions used in the protocol were 30 s at 94°C followed by 45 cycles at 94°C for 5 s, 60°C for 30 s, and the dissociation stage. Gene expression levels were analyzed relative to the level of the GAPDH gene transcript. The primers used were as follow (see Table 1).

**TABLE 1:** PCR primer sequences.

**Gene**	**Primer sequences**
Nrf2	F:5′-GCCTTACTCTCCCAGTGAATAC-3′
	R:5′-CTCCCAAATGGTGCCTAAGA-3′
Trx-1	F: 5′-GGAATGGTGAAGCAGATCGAG-3′
	R:5′-ACGCTTAGACTAATTCATTAAT-3′
GAPDH	F: 5′-GAGCCCTTCCACAATGCCAAAGTT-3′
	R:5′-TGTGATGGGTGTGAACCACGAGAA-3′

*F, forward primer; R, reverse primer.*

### Biochemical Analysis

Determination of hippocampal tissue catalase (CAT), superoxide dismutase (SOD), malondiadehyde (MDA) and glutathione peroxidase (GSH-PX) activities. According to the manufacturer’s instructions, the collected organs were homogenized in 50 mM phosphate buffer cooled to 4°C containing K2HPO4 + KH2PO4 + 0.1 mM ethylenediaminetetraacetic acid (EDTA) (pH, 7.0) + 0.1% BSA and then centrifuged. The obtained brain supernatants were used for further investigations. The absorbance of each well was determined by a microplate reader, and the activity was calculated according to a formula. The SOD, MDA, GSH-PX and CAT activities were measured. The activities of the studied antioxidant enzymes in tissue extracts were calculated in U/mg of protein.

### Statistical Analysis

All values are expressed as the mean ± standard deviation (SD). The statistical analyses were completed with one-way analysis of variance (ANOVA) followed by Tukey’s *post hoc* test by GraphPad Prism 5.0 software (GraphPad Software, Inc., La Jolla, CA, United States). Differences were considered significant at *p* < 0.05.

## Results

### Blood Glucose Changes in Each Group of Mice

There were no significant differences in mobility, body weight, or blood glucose levels between the mice in each group prior to STZ modeling. Compared with the WT-NC group, blood glucose in the WT-DM group was significantly higher (*p* < 0.01), and compared with the TG-NC group, blood glucose in the TG-DM group was significantly higher (*p* < 0.05) after 3 days of STZ injection, and the blood glucose was persistently high (see Table 2).

**TABLE 2:** Blood glucose test results for each group of mice (mmol/L).

	**WT-NC**	**WT-DM**	**TG-NC**	**TG-DM**
1 day	6.83 ± 1.28	6.77 ± 0.87	6.23 ± 1.76	6.80 ± 0.32
4 days	6.50 ± 0.56	21.56 ± 2.35**	6.38 ± 1.43	15.93 ± 1.12^#^
4 weeks	7.38 ± 0.38	26.7 ± 2.31**	7.85 ± 0.61	21.44 ± 0.12^##^
8 weeks	7.62 ± 1.19	25.68 ± 0.65**	7.95 ± 0.56	17.17 ± 0.68^##^
12 weeks	7.75 ± 1.56	25.89 ± 3.19**	7.62 ± 2.36	20.14 ± 1.22^##^

**p < 0.01, comparison between the WT-NC group and the WT-DM group; #*p* < 0.05, ##*p* < 0.01, comparison between the TG-NC group and the TG-DM group.

### High Expression of Trx-1 Gene in Transgenic Mice Improves Spatial Learning and Memory

**FIGURE 1:**
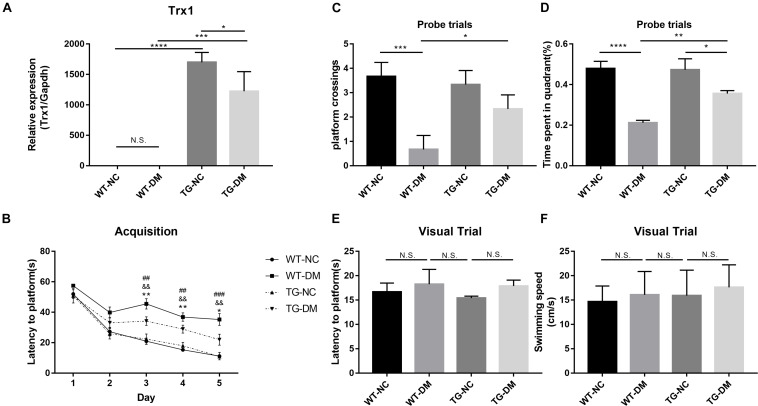
Trx-1 mRNA levels in the hippocampal tissues of the mice and neurobehavioral analysis of Trx-1 transgenic mice. **(A)** Real-time quantitative PCR of Trx-1 mRNA levels in mouse hippocampal tissue. **(B)** Latency to platform, ##*p* < 0.01: WT-NC *vs* WT-DM; &&*p* < 0.01: WT-DM *vs* TG-DM; *p < 0.05, **p < 0.01: TG-NC *vs* TG-DM. **(C)** Platform crossing (probe trial), **(D)** Time spent in the target quadrant (probe trial). **(E)** Latency to platform (visual trial). **(F)** Swimming speed. Tukey’s *post hoc* test for one-way ANOVA was used to determine Trx-1 mRNA levels. For each group, *n* = 6. The vertical lines represent the standard error of the mean. *p < 0.05, **p < 0.01, ***p < 0.001, and ****p < 0.0001.

In the present experiment, we aimed to enhance the anti-ERS and antioxidative stress effects of Trx-1 by transgenically increasing the expression of the Trx-1 gene in mice. Real-time PCR was used to detect Trx-1. The results showed that the Trx-1 gene was upregulated in both the TG-NC group and TG-DM group compared with the WT-NC and WT-DM groups (*p* < 0.0001 and *p* < 0.001, respectively), and upregulated in TG-NC group compared with the TG-DM group (*p* < 0.05) (see Figure 1A).

To evaluate whether the DM mice exhibited cognitive impairment and whether Trx-1 overexpression positively affected this impairment, we used the Morris water maze test, a well-established test for spatial learning and memory, to assess spatial learning and memory in each group of mice. We found that there was a downward trend in escape latency on the second day in all groups, and there were no significant differences among the groups. From the third to the fifth day, except for the WT-DM group, the escape latency of the other three groups still showed a downward trend; however, the WT-DM group had a smaller trend than the other two groups. There were significant differences in the escape latency between the WT-DM group and the WT-NC group (*p* < 0.01, *p* < 0.01, and *p* < 0.01, respectively), as well as the TG-DM group (*p* < 0.01, *p* < 0.01, and *p* < 0.001, respectively). In addition, the TG-NC and TG-DM groups also showed differences on the next 3 days (*p* < 0.01, *p* < 0.01 and *p* < 0.05, respectively) (see Figure 1B). In the ascending platform test, which was carried out 24 h later, we found that the platform crossings and the time spent in the target quadrant in the WT-DM group were significantly reduced compared with those of the WT-NC group (*p* < 0.001, *p* < 0.0001, respectively) and the TG-DM group (*p* < 0.05, *p* < 0.01, respectively) (see Figures 1C,D). Finally, in the swimming speed and visual platform tests, there were no significant differences among the groups (see Figures 1E,F).

### Trx-1 Alleviates the Hippocampal Neurodegeneration and Hippocampal Apoptosis Induced by DM

**FIGURE 2:**
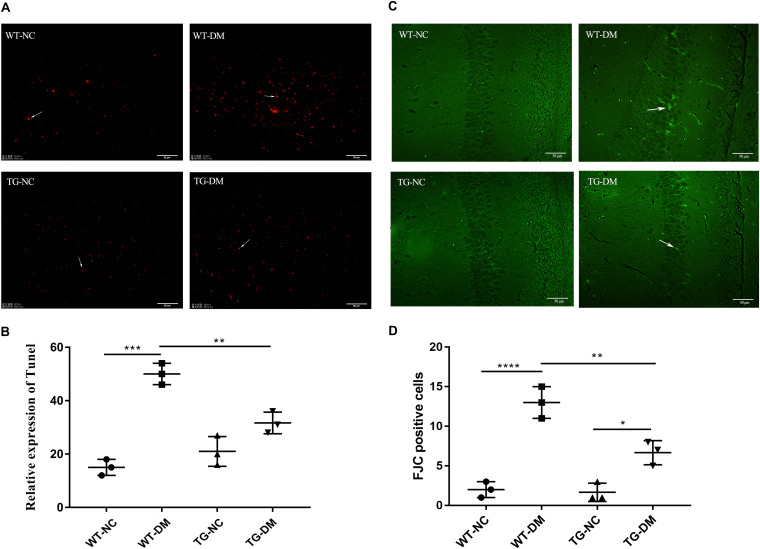
Trx-1 significantly inhibits DM-induced apoptosis and neural degeneration in the hippocampal region. **(A)** Positive cells with a red fluorescence signal (white arrows) were present in the hippocampal as determined by the TUNEL assay. **(B)** Quantitative statistical analysis of TUNEL-positive cells. **(C)** Representative images of FJC-positive staining (white arrows) in the hippocampal regions. **(D)** Quantitative statistical analysis of FJC-positive cells. Tukey’s *post hoc* test for one-way ANOVA used for TUNEL test. For each group, *n* = 6. The vertical lines represent the standard error of the mean. *p < 0.05, **p < 0.01, ***p < 0.001, ****p < 0.0001.

We evaluated the apoptosis of neurons by the TUNEL assay to verify the damage to neurons in the hippocampus induced by DM. We found that the number of TUNEL-positive cells with red fluorescence signals was much higher in the STZ-treated transgenic and non-transgenic DM mice than in the control DM mice without STZ treatment (see Figure 2A). Quantitative analysis showed that the number of TUNEL-positive cells was significantly increased in the WT-DM group compared with the WT-NC group (*p* < 0.001), but in the TG-DM group, the number of positive cells was significantly decreased compared with the WT-DM group (*p* < 0.01) (see Figure 2B).

Fluoro-JadeC (FJC) staining was used to label damaged cells to further determine the severity of neuronal degeneration. The number of FJC-positive cells in the hippocampal regions of the WT-DM group was significantly increased compared with that of the WT-NC group (*p* < 0.0001), and the number of FJC-positive cells in the hippocampal CA1 and CA3 regions of the TG-DM group was significantly decreased compared with that of the WT-DM group (*p* < 0.01) (see Figures 2C,D).

### Trx-1 Reduces Aβ Deposition and the Immunocontent of the AD-Related Protein p-Tau in DM Mice

**FIGURE 3:**
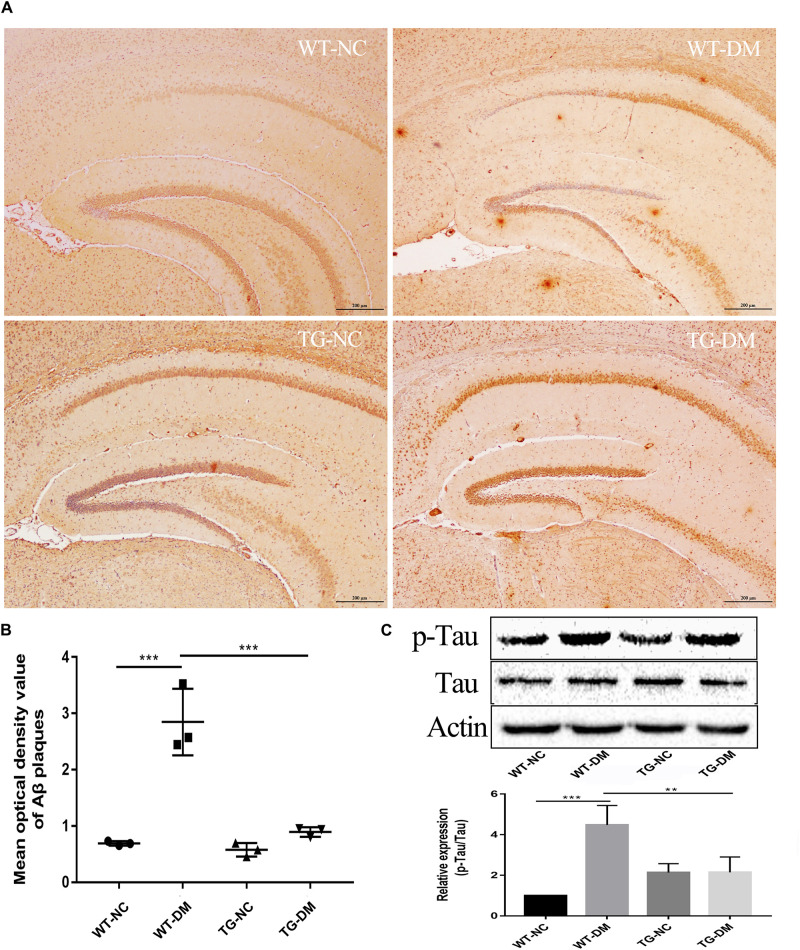
Trx-1 reduced Aβ burden and Tau phosphorylation in DM mice. **(A)** Representative images of Aβ deposits in the hippocampus. **(B)** Quantitative statistical analysis of Aβ density value in the hippocampus. **(C)** Representative Western blot images and analysis of Tau and p-Tau. Tukey’s *post hoc* test for one-way ANOVA used for Aβ density value and Western blot immunocontent. For each group, *n* = 6. The vertical lines represent the standard error of the mean. **p < 0.01, ***p < 0.001.

To examine AD-like pathological changes in DE and the effect of Trx-1 on the pathological changes, the formation of Aβ plaques in the brain and the load of amyloid plaques in mice were quantified by immunohistochemistry (IHC) with antibodies against Aβ. Through observation and calculation of the density of Aβ-positive plaques in the hippocampus, we found that Aβ plaques were present in the WT-DM and TG-DM groups but not in the other two groups (see Figure 3A), and the Aβ plaque density in the TG-DM group was significantly lower than that in the WT-DM group (*p* < 0.001) (see Figure 3B). Western blot analysis was performed to detect the immunocontent of Tau and p-Tau. The results showed that the p-Tau immunocontent levels were significantly increased in the WT-DM group compared with the WT-NC group (*p* < 0.001). However, the p-Tau immunocontent was decreased in the TG-DM group compared with the WT-DM group (*p* < 0.01) (see Figure 3C).

### Trx-1 Affects DM-Induced ERS Marker Proteins and Inhibits DM-Induced Activation of PERK Pathway-Associated Proteins

**FIGURE 4:**
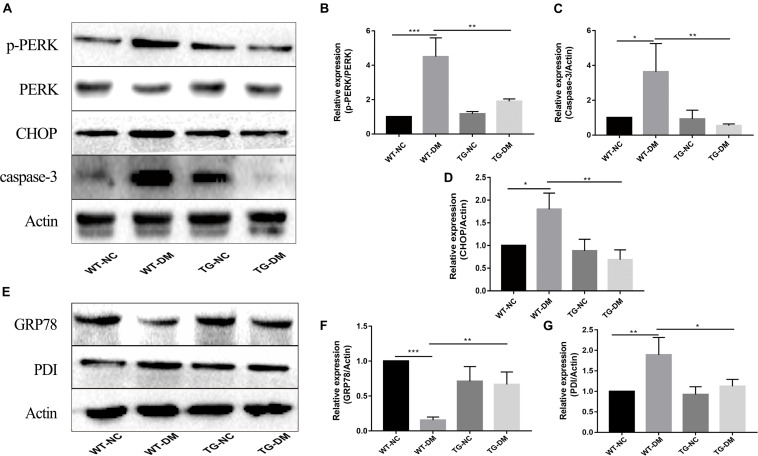
Effects of DM and Trx-1 on the protein immunocontent of GRP78 and PDI and changes in the activation of PERK, CHOP and caspase-3 in the PERK receptor-related signaling pathway in DM mice. **(A)** Representative protein expression bands of PERK, p-PERK, CHOP and caspase-3. **(B)** Representative Western blot analysis of p-PERK/PERK. **(C)** Representative Western blot analysis of caspase-3. **(D)** Representative Western blot analysis of CHOP. **(E)** Representative protein expression bands of GRP78 and PDI. **(F)** Representative Western blot analysis of GRP78. **(G)** Representative Western blot analysis of PDI. Tukey’s *post hoc* test for one-way ANOVA was used to analyze the Western blot immunocontent. For each group, *n* = 6. The vertical lines represent the standard error of the mean. *p < 0.05, **p < 0.01, and ***p < 0.001.

To investigate the mechanism by which the ERS PERK receptor-related pathway is involved in the effects of Trx-1, Western blot analysis was performed to detect the immunocontent of PERK, p-PERK, CHOP and caspase-3. The results showed that the p-PERK, CHOP and caspase-3 immunocontent levels were significantly increased in the WT-DM group compared with the WT-NC group (*p* < 0.001, *p* < 0.05, and *p* < 0.05, respectively) (see Figures 4A–D). However, the p-PERK, CHOP and caspase-3 immunocontent levels were significantly decreased in the TG-DM group compared with the WT-DM group. (*p* < 0.01, *p* < 0.01, *p* < 0.01, respectively) (see Figures 4A–D).

GRP78 and PDI, both ER-resident chaperones, are markers of ERS that play crucial roles in the dynamic regulation of ER homeostasis (Jui-Ching et al., 2008; Li-Rong et al., 2013). We further examined the effect of Trx-1 on DM-induced ERS. The results showed that Trx-1 inhibited the DM-induced decrease in GRP78 levels (see Figures 4E,F). Moreover, the DM-induced activation of PDI expression was inhibited by Trx-1 (see Figures 4E,G). These data suggest that Trx-1 attenuated DM-induced ERS by enhancing the expression of GRP78 and inhibiting the expression of PDI.

### Trx-1 Inhibits DM-Induced Activation of IRE1α Pathway-Associated Proteins

**FIGURE 5:**
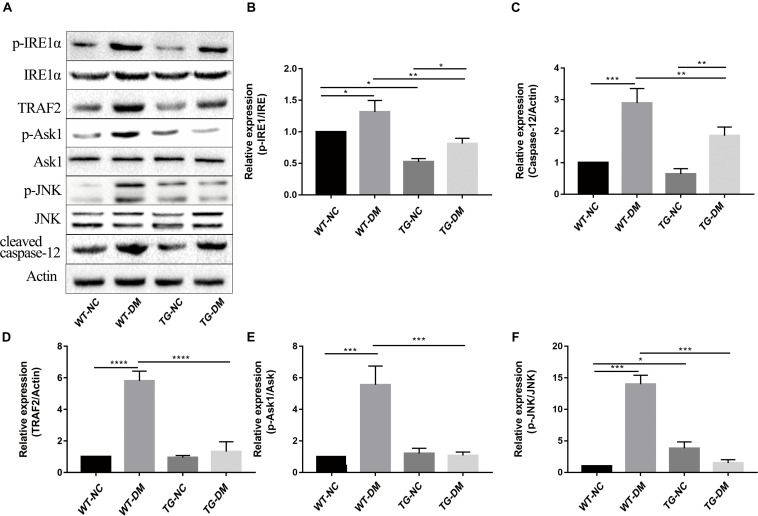
Changes in the activation of IRE1α, TRAF2, ASK1, JNK, and caspase-12 in the IRE1α receptor-related pathway signaling pathway in DM mice. **(A)** Representative protein expression bands of IRE1α, TRAF2, ASK1, JNK, and caspase-12. **(B)** Representative Western blot analysis of p-IRE1α/IRE1α. **(C)** Representative Western blot analysis of caspase-12. **(D)** Representative Western blot analysis of TRAF2. **(E)** Representative Western blot analysis of p-ASK1/ASK1. **(F)** Representative Western blot analysis of p-JNK/JNK. Tukey’s *post hoc* test for one-way ANOVA was used to analyze the Western blot immunocontent. For each group, *n* = 6. The vertical lines represent the standard error of the mean. *p < 0.05, **p < 0.01, ***p < 0.001, and ****p < 0.0001.

We next investigated the mechanism by which the ERS IRE1α receptor-related pathway is involved in the effects of Trx-1. Western blot analysis was performed to detect the immunocontent of IRE1α, p-IRE1α, TRAF2, ASK1, p-ASK1, JNK, p-JNK, and caspase-12. The results showed that the p-IRE1α, TRAF2, p-ASK1, p-JNK, and caspase-12 immunocontent levels were significantly increased in the WT-DM group compared with the WT-NC group. (*p* < 0.05, *p* < 0.0001, *p* < 0.001, *p* < 0.001, and *p* < 0.001, respectively) (see Figures 5A–F). However, the abovementioned protein immunocontent levels were significantly decreased in the TG-DM group compared with the WT-DM group (*p* < 0.01, *p* < 0.0001, *p* < 0.001, *p* < 0.001, and *p* < 0.01, respectively) (see Figures 5A–F).

### Trx-1 Upregulates the Gene and Protein Levels of Nrf2, Antioxidant Defense Parameters and Decreases Phosphorylated NFκB Levels in the Hippocampus of DM Mice

**FIGURE 6:**
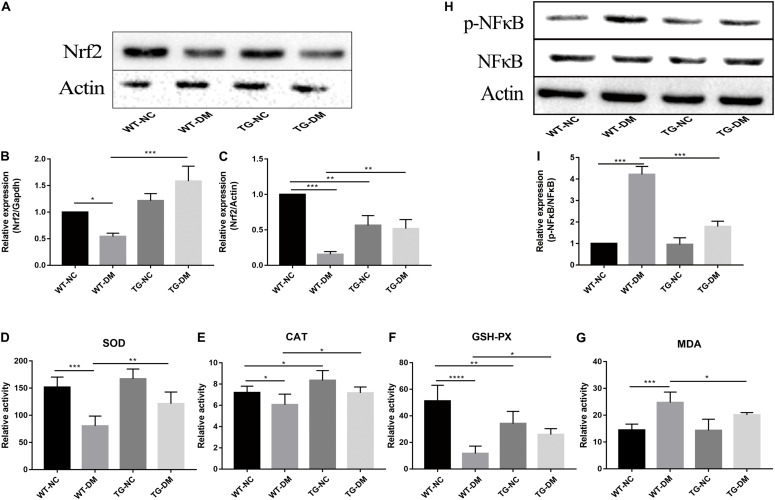
Effects of Trx-1 on the gene and protein levels of Nrf2, the activation of NFκB and antioxidant defense parameters. **(A)** Representative protein expression bands of Nrf2. **(B)** Real-time quantitative PCR of Nrf2 mRNA levels in mice hippocampal tissue. **(C)** Representative Western blot analysis of Nrf2. **(D)** Antioxidant defense parameter: SOD activity analysis. **(E)** Antioxidant defense parameter: CAT activity analysis. **(F)** Antioxidant defense parameter: GSH-PX activity analysis. **(G)** Antioxidant defense parameter: MDA activity analysis. **(H)** Representative bands of the expression of NFκB. **(I)** Representative Western blot analysis of p-NFκB/NFκB. Tukey’s *post hoc* test for one-way ANOVA was used to analyze the mRNA levels, Western blot immunocontent, and antioxidant defense parameters. For each group, *n* = 6. The vertical lines represent the standard error of the mean. *p < 0.05, **p < 0.01, ***p < 0.001, and ****p < 0.0001.

Changes in the immunocontent of Nrf2 partly reflected the degree and development of antioxidant stress. Real-time PCR was used to detect Nrf2. The results showed that both the Nrf2 gene and immunocontent were downregulated in the WT-DM group compared with the WT-NC group (*p* < 0.001 and *p* < 0.05, respectively) and upregulated in the TG-DM group compared with the WT-DM group (*p* < 0.01 and *p* < 0.001, respectively) (see Figures 6A–C).

Statistically significant decreases in the activities of SOD, CAT and GSH-PX were observed in the WT-DM group compared with the WT-NC group (*p* < 0.001, *p* < 0.05 and *p* < 0.0001, respectively), but the MDA activities in the WT-DM group was significantly increased compared with it in the WT-NC group (*p* < 0.001). Furthermore, the SOD, CAT, and GSH-PX activities in the TG-DM group were significantly increased compared with those in the WT-DM group (*p* < 0.01, *p* < 0.05, and *p* < 0.05, respectively), but statistically significant decreases in the activities of MDA was observed in the TG-DM group compared with the WT-DM group (*p* < 0.05), and CAT, GSH-PX were also significantly increased in the TG-NC group compared with the WT-NC group (*p* < 0.01 and *p* < 0.01, respectively) (see Figures 6D–G).

Finally, we tested the activation of NFκB, and the results showed that the immunocontent of phosphorylated NFκB was significantly increased in the WT-DM group compared with the WT-NC group (*p* < 0.001) and significantly decreased in the TG-DM group compared with the WT-DM group (*p* < 0.001) (see Figures 6H,I).

## Discussion

DM is one of the most common diseases affecting humans. The pathological changes of DE are similar to those of AD (Sima, 2010). Moreover, epidemiological studies have shown that diabetic patients have a higher risk of developing AD than healthy people, and there are some common mechanisms and interactions between AD and DE (MacKnight et al., 2002; Fukazawa et al., 2013). These mechanisms have not been fully elucidated. In this study, we explored whether DE caused by DM has AD-like pathological changes. We found that Trx-1 overexpression in transgenic mice alleviated the STZ-induced dysfunction in learning and memory and reduced cell degeneration and apoptosis. This process was achieved by the upregulated Trx-1 exerting resistance to ERS and OS.

We also observed Aβ deposition only in the hippocampus of diabetic and transgenic mice, which supported the conclusion that diabetes is a risk factor for AD. The microtubule-associated protein Tau is a cytoskeletal protein mainly expressed by neurons and is primarily located in the axonal chamber (MacKnight et al., 2002). One of the characteristics of AD is the accumulation of misfolded Tau protein in neurons, which results in neurofibrillary tangle production and neuronal cell dysfunction and death (Ittner et al., 2010). As a molecular chaperone induced by ERS, PDI has been found to coexist with neurofibrillary tangles in the brains of patients with AD and is thought to prevent neurotoxicity associated with ERS and protein misfolding (Li-Rong et al., 2013). It has been reported that PDI and Tau can form a 1:1 complex to prevent the abnormal aggregation of Tau under physiological conditions (Li-Rong et al., 2013). In our study, we found that the immunocontent of p-Tau and PDI in the hippocampus of diabetic mice was significantly increased, indicating that diabetes can promote the activation of Tau, and the increase in PDI also indicates that diabetes can exacerbate ERS (see Figures 3, 4).

**FIGURE 7:**
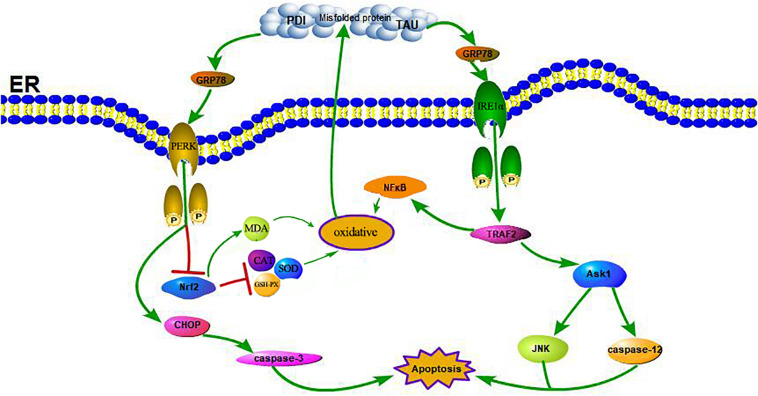
Schematic illustration of the DM-induced ERS and OS. DM induced apoptosis through activation of the signaling pathway IRE1α, TRAF2, ASK1, JNK, caspase-12, and the pathway PERK, CHOP, caspase-3. Meanwhile, the reduction of Nrf2 induced by activation of PERK and the increase of NFκB induced by activation of TRAF2 contribute to enhance oxidative stress, further lead to a increase of PDI, p-TAU and accumulation of misfolded proteins which in turn increased the activation of the receptor IRE1α and PERK.

A large number of studies have shown that ERS can contribute to neuronal death in AD (Gerakis and Hetz, 2018). GRP78 is a marker of ERS; under non-stress conditions, it binds to three ERS receptors, IRE1α, PERK, and ATF6, and inhibits their activation (Yi et al., 2017). In response to ERS, GRP78 dissociates from these transmembrane proteins, causing them to activate and trigger the UPR (see Figure 7). Moreover, some studies have shown that the upregulation of GRP78 may decrease with time, such as in the hippocampus of STZ-treated diabetic mice and in islets and NIT-1 cells treated with STZ at different stages, in which the expression of GRP78 protein increased at first and then decreased (Wang et al., 2007; Cao et al., 2014; Zhao et al., 2015). The change in GRP78 expression partly reflects the development process of ERS. In this study, the expression of GRP78 in the WT-DM group decreased significantly (see Figure 4), indicating that the development of ERS had reached a later stage, which may also be key to AD-like changes.

**FIGURE 8:**
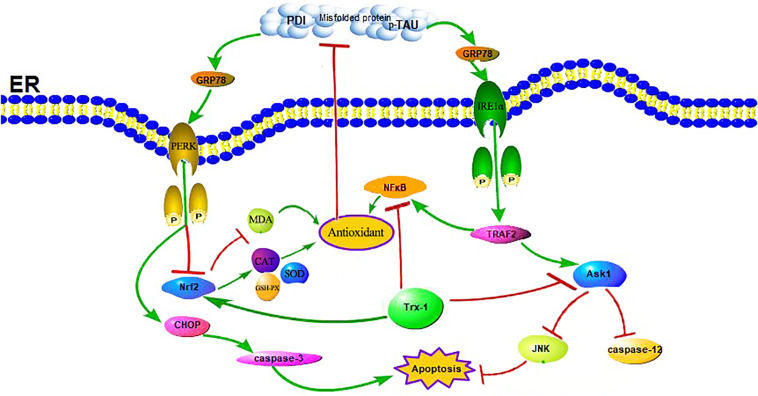
Schematic illustration of the role of Trx-1 in regulating DM-induced ERS and OS. On the one hand, the upregulation of Trx-1 increased Nrf2 and inhibited NFκB, leading to the increase of anti-oxidant enzymes and the decrease of oxidases to exert antioxidant stress and anti-endoplasmic reticulum stress, which reduced misfold protein production. On the other hand, the upregulation of Trx-1 inhibited ASK1 phosphorylation, decreased the expression of JNK and caspase-12, further exerted antiapoptotic effects.

Trx-1, an important antioxidant protein, not only protects cell activity by exerting its oxidoreductase activity through conserved Cys32 and Cys35, reducing oxidized proteins through thiol disulfide exchange reactions (Nagarajan et al., 2016), but also plays an important role in cell signal transduction and gene transcription (Bai et al., 2003). Other studies have shown that Trx-1 overexpression in mice can significantly prolong survival during septicemia by inhibiting ERS (Bai et al., 2007; Chen et al., 2016). Our previous studies have shown that sulforaphane (SF) can protect diabetic hippocampal neurons by upregulating Trx-1 expression (Tang et al., 2020). Indeed, in animal model of STZ-induced diabetes in mice, ROS were increased and Trx-1 activity was significantly decreased without a change in expression or protein levels in the diabetic animals in comparison to untreated littermates (Schulze et al., 2004). One may speculate that this glucose-dependent upregulation of Txnip also disturbs the Trx-1/ASK-1 axis, since the enhanced inhibitory binding of Txnip to Trx-1 releases ASK-1, which then induces endothelial cells (EC) apoptosis. Therefore, the regulation of Trx-1 in the pathogenesis of diabetes may also be accomplished by modulating ERS (Zschauer et al., 2013; see Figure 8). It has also been reported that the expression of Trx-1 is reduced in AD, a fact that suggests that the reduced level of Trx-1 may be related to the neurodegenerative mechanisms of AD: its reduction may contribute to the neurodegenerative mechanisms of AD, which may be related to the interaction of Trx-1 with ASK1 (Akterin et al., 2006). And our results also found that the expression level of Trx-1 was reduced in the hippocampus of mice in the TG-DM group compared with the TG-NC group (see Figure 1) and which is consistent with the results reported above, and the extent of ASK1 phosphorylation was also attenuated in TG-DM compared with WT-DM, Trx-1 may play a key role in AD through the regulation of ASK1 (see Figure 5).

In ERS signaling pathways, p-IRE1α is thought to initiate apoptosis and is significantly related to cell death (Kim et al., 2017). Activated IRE1α has been shown to recruit the adaptive molecules TRAF2 and ASK1 to form a complex, activate caspase-12 on the ER and activate JNK to initiate apoptosis (see Figure 7; Szegezdi et al., 2006). Trx-1 has been reported to act as an endogenous inhibitor of ASK1 (Saitoh et al., 1998); when Trx-1 is reduced it binds ASK1. Thus, overexpression of Trx-1 may repress JNK activation by suppressing ASK1 (see Figure 8). An increase in the ERS has been shown to result in the activation of NFκB. The IRE1α-TRAF2 complex recruits I kappa B (IκB) kinase, which phosphorylates IκB and leads to NFκB degradation, leading to the exacerbation of inflammatory reactions and OS (Zha et al., 2015). Recent study demonstrated that CHOP could also modulate NFκB activation by decreasing IκB degradation and p65 translocation (see Figure 7). We further examined the expressions of NFκB in the present study. We demonstrated that Trx-1 overexpression inhibited the NFκB activation in DE and AD induced DM (Figure 6). This data suggested that Trx-1 overexpression was able to inhibit the NFkB signaling pathway (see Figure 8). On the other hand, GRP78 induces the PERK pathway and activates the downstream expression of CHOP and caspase-3 to initiate apoptosis (see Figure 7; Sankrityayan et al., 2019). All of these factors may eventually lead to neuronal apoptosis, and IRE1α, TRAF2, ASK1, JNK, caspase-12, PERK, CHOP, and caspase-3 were activated in this study, indicating that ERS was induced by diabetes (see Figure 4).

Furthermore, Nrf2 is considered to be a substrate of PERK. The phosphorylation of PERK can trigger conformational changes in the Nrf2 protein and trigger the dissociation of the Keap1-Nrf2 complex and the translocation of Nrf2 into the nucleus, thus upregulating the expression of antioxidant genes (Zhu et al., 2015), and this activates the transcription of more than 200 stress/antioxidant enzymatic genes such as glutathione, glutathione reductase, thioredoxin, thioredoxin-S-transferase, and catalase (Thimmulappa et al., 2002; Aldana et al., 2003; Datta et al., 2017; Periyasamy and Shinohara, 2017). Therefore, there is a clear correlation between OS and ERS through the PERK/Nrf2 pathway (see Figure 7; Thummayot et al., 2016). Thus, interfering with the Nrf2 response leads to the accumulation of damaged proteins in the ER, which leads to PERK-dependent apoptosis, suggesting that OS can induce ERS, and excessive ERS can also cause or exacerbate OS through PERK/Nrf2 crosstalk (Hayashi et al., 2005). In this study, we also confirmed that at both the protein and gene levels, the expression of Nrf2 in the WT-DM group was lower than that in the WT-NC group, and other oxidative stress-related indicators, such as SOD, CAT and GSH-PX, showed the same trend as Nrf2, but the MDA showed the reverse trend as Nrf2 (see Figure 6). SOD is an important antioxidant enzyme, which can effectively scavenge free radicals and thus resist the damage of oxygen free radicals to cells (Wang et al., 2018). Therefore, SOD plays a very important role in the balance of oxidation and antioxidation, and its activity indirectly reflects the ability of the body to scavenge oxygen free radicals. MDA is the product of lipid peroxidation in brain tissue, especially during brain injury, a large amount of oxygen free radicals can make lipid peroxidation, so the increase of MDA content further indicates that oxidative stress is active in diabetes, thus proving the importance of oxidative stress in the occurrence and development of DE (Wang et al., 2018). These results showed that in the later stages of ERS, ERS and OS induce and promote each other. Therefore, in this study, in Trx-1 transgenic mice (both transgenic control and transgenic diabetic mice), the elevated Nrf2 levels were achieved through the upregulation of Trx-1. In turn, the elevated Nrf2 affected SOD, CAT, MDA and GSH-PX, reversing the decrease in SOD, CAT and GSH-PX activity and increase in MDA, thereby counteracting DM-induced ERS and OS and reducing PDI as well as misfolded protein production, the degree of neuronal apoptosis and degeneration and the production of Aβ plaques, indicating that Trx-1 has antidiabetic effects and opposes AD-like changes (see Figures 4, 6). These could be potential mechanisms by which the decline in learning and memory induced by diabetes was inhibited in transgenic diabetic mice.

In summary, DE causes AD-like pathological changes, and the Trx-1 transgene alleviated these pathological changes and improved learning and memory function in DE. The upregulation of Trx-1 increased Nrf2 and inhibited NFκB and ASK1 phosphorylation, which exerted anti-endoplasmic reticulum stress, antioxidant stress, and antiapoptotic effects (see Figure 8). Therefore, Trx-1 may be a potential target for regulating ERS and OS in DE and AD.

## Data Availability Statement

The original contributions presented in the study are included in the article/Supplementary Material, further inquiries can be directed to the corresponding author/s.

## Ethics Statement

The animal study was reviewed and approved by All procedures were performed in accordance with the Guide for the Care and Use of Laboratory Animals of the National Institutes of Health (NIH), and all protocols were approved by the Institutional Animal Care and Use Committee of Dalian Medical University.

## Author Contributions

All authors listed have made a substantial, direct and intellectual contribution to the work, and approved it for publication.

## Conflict of Interest

The authors declare that the research was conducted in the absence of any commercial or financial relationships that could be construed as a potential conflict of interest.

## References

[B1] Akterin S. Cowburn R. F. Miranda-Vizuete A. Jiménez A. Bogdanovic N. Winblad B. (2006). Involvement of glutaredoxin-1 and thioredoxin-1 in beta-amyloid toxicity and Alzheimer’s disease. *Cell Death Differ.* 13 1454–1465. 10.1038/sj.cdd.4401818 16311508

[B2] Aldana J. P. A. Marcovich R. Singhal P. Reddy K. Morgenstern N. El-Hakim A. (2003). Immune response to laparoscopic bowel injury. *J. Endourol.* 17 317–322. 10.1089/089277903322145503 12885358

[B3] Almanza A. Carlesso A. Chintha C. Creedican S. Doultsinos D. Leuzzi B. (2018). Endoplasmic reticulum stress signalling - from basic mechanisms to clinical applications. *FEBS J.* 286 241–278. 30027602 10.1111/febs.14608 PMC7379631

[B4] Asano T. Fujishiro M. Fujishiro M. Nakatsu Y. Yoneda M. Kamata H. (2007). Role of phosphatidylinositol 3-kinase activation on insulin action and its alteration in diabetic conditions. *Biol. Pharmaceutic. Bull.* 30:1610. 10.1248/bpb.30.1610 17827708

[B5] Aubert L. Pichierri S. Hommet C. Camus V. Berrut G. de Decker L. (2015). Association between comorbidity burden and rapid cognitive decline in individuals with mild to moderate Alzheimer’s disease. *J. Am. Geriatr. Soc.* 63 543–547. 10.1111/jgs.13314 25752337

[B6] Bai J. Nakamura H. Kwon Y. W. Hattori I. Yamaguchi Y. Kim Y. C. (2003). Critical roles of thioredoxin in nerve growth factor-mediated signal transduction and neurite outgrowth in PC12 cells. *J. Neurosci.* 23 503–509. 10.1523/jneurosci.23-02-00503.2003 12533610 PMC6741868

[B7] Bai J. Nakamura H. Kwon Y. W. Tanito M. Ueda S. Tanaka T. (2007). Does thioredoxin-1 prevent mitochondria- and endoplasmic reticulum-mediated neurotoxicity of 1-methyl-4-phenyl-1,2,3,6-tetrahydropyridine? *Antioxid Redox Signal.* 9 603–608. 10.1089/ars.2006.1513 17465883

[B8] Biessels G. J. ter Laak M. P. Hamers F. P. Gispen W. H. (2002). Neuronal Ca2+ disregulation in diabetes mellitus. *Eur. J. Pharmacol.* 447 201–209. 10.1016/s0014-2999(02)01844-7 12151012

[B9] Cao Y. Hao Y. Li H. Liu Q. Gao F. Liu W. (2014). Role of endoplasmic reticulum stress in apoptosis of differentiated mouse podocytes induced by high glucose. *Int. J. Mol. Med.* 33 809–816. 10.3892/ijmm.2014.1642 24503896 PMC3976130

[B10] Chen G. Chen G. Li X. Huang M. Li M. Zhou X. (2016). Thioredoxin-1 increases survival in sepsis by inflammatory response through suppressing endoplasmic reticulum stress. *Shock* 46 67–74. 10.1097/shk.0000000000000570 27299588

[B11] Clodfelder-Miller B. Zmijewska A. A. Johnson G. V. Jope R. S. (2006). Tau is hyperphosphorylated at multiple sites in mouse brain in vivo after streptozotocin-induced insulin deficiency. *Diabetes* 55 3320–3325. 10.2337/db06-0485 17130475 PMC1851885

[B12] Dan L. Korhonen L. Eriksson O. Kõks S. (2017). Recent insights into the role of unfolded protein response in er stress in health and disease. *Front. Cell Dev. Biol.* 5:48. 10.3389/fcell.2017.00048 28540288 PMC5423914

[B13] Datta S. Cano M. Ebrahimi K. Wang L. Handa J. T. (2017). The impact of oxidative stress and inflammation on RPE degeneration in non-neovascular AMD. *Prog. Retinal Eye Res.* 60 201–218. 10.1016/j.preteyeres.2017.03.002 28336424 PMC5600827

[B14] Díaz-Gerevini G. T. Repossi G. Dain A. Tarres M. C. Das U. N. Eynard A. R. (2014). Cognitive and motor perturbations in elderly with longstanding diabetes mellitus. *Nutrition* 30 628–635. 10.1016/j.nut.2013.11.007 24800665

[B15] Fukazawa R. Hanyu H. Sato T. Shimizu S. Koyama S. Kanetaka H. (2013). Subgroups of Alzheimer’s disease associated with diabetes mellitus based on brain imaging. *Demen. Geriatr. Cogn. Disord.* 35 280–290. 10.1159/000348407 23594859

[B16] Gerakis Y. Hetz C. (2018). Emerging roles of ER stress in the etiology and pathogenesis of Alzheimer’s disease. *FEBS J.* 285 995–1011. 29148236 10.1111/febs.14332

[B17] Glory A. Averill-Bates D. A. (2016). The antioxidant transcription factor Nrf2 contributes to the protective effect of mild thermotolerance (40°C) against heat shock-induced apoptosis. *Free Radic. Biol. Med.* 99 485–497. 10.1016/j.freeradbiomed.2016.08.032 27591796

[B18] Han C. Wang L. Wang C. (2009). Domain a’ of protein disulfide isomerase plays key role in inhibiting α-synuclein fibril formation. *Cell Stress Chaperones* 15 415–421. 10.1007/s12192-009-0157-2 19960284 PMC3082648

[B19] Hayashi T. Saito A. Okuno S. Ferrand-Drake M. Dodd R. L. Chan P. H. (2005). Damage to the endoplasmic reticulum and activation of apoptotic machinery by oxidative stress in ischemic neurons. *J. Cereb. Blood Flow Metab.* 25 41–53. 10.1038/sj.jcbfm.9600005 15678111

[B20] Holmgren A. (1985). Thioredoxin. *Annu. Rev. Biochem.* 54 237–271. 3896121 10.1146/annurev.bi.54.070185.001321

[B21] Ittner L. M. Ke Y. D. Delerue F. Bi M. Gladbach A. van Eersel J. (2010). Dendritic function of tau mediates amyloid-beta toxicity in Alzheimer’s disease mouse models. *Cell* 142 387–397. 10.1016/j.cell.2010.06.036 20655099

[B22] Jin J. K. Blackwood E. A. Azizi K. Thuerauf D. J. Fahem A. G. Hofmann C. (2016). ATF6 decreases myocardial ischemia/reperfusion damage and links er stress and oxidative stress signaling pathways in the heart. *Circ. Res.* 120:862. 10.1161/circresaha.116.310266 27932512 PMC5336510

[B23] Matés JM. Sánchez-Jiménez FM. (2000). Role of reactive oxygen species in apoptosis: implications for cancer therapy. *Int. J. Biochem. Cell Biol.* 32 157–170. 10.1016/s1357-2725(99)00088-6 10687951

[B24] Jui-Ching C. Wu M. L. Huang K. C. Lin W. W. (2008). HMG-CoA reductase inhibitors activate the unfolded protein response and induce cytoprotective GRP78 expression. *Cardiovasc. Res.* 80 138–150. 10.1093/cvr/cvn160 18556704

[B25] Kim J. Song H. Heo H. R. Kim J. W. Kim H. R. Hong Y. (2017). Cadmium-induced ER stress and inflammation are mediated through C/EBP–DDIT3 signaling in human bronchial epithelial cells. *Exp. Mol. Med.* 49:e372. 10.1038/emm.2017.125 28860664 PMC5628270

[B26] Lim J. L. Wilhelmus M. M. de Vries H. E. Drukarch B. Hoozemans J. J. van Horssen J. (2014). Antioxidative defense mechanisms controlled by Nrf2: state-of-the-art and clinical perspectives in neurodegenerative diseases. *Arch. Toxicol.* 88 1773–1786. 10.1007/s00204-014-1338-z 25164826

[B27] Li-Rong X. Liu X. L. Chen J. Liang Y. (2013). Protein disulfide isomerase interacts with tau protein and inhibits its fibrillization. *PLoS One* 8:e76657. 24098548 10.1371/journal.pone.0076657 PMC3788760

[B28] Luo F. C. Zhou J. Qi L. Wang S. D. Nakamura H. Yodoi J. (2012). Induction of endoplasmic reticulum stress and the modulation of thioredoxin-1 in formaldehyde-induced neurotoxicity. *NeuroToxicology* 33 290–298. 10.1016/j.neuro.2012.02.004 22342837

[B29] Lupachyk S. Watcho P. Stavniichuk R. Shevalye H. Obrosova I. G. (2013). Endoplasmic reticulum stress plays a key role in the pathogenesis of diabetic peripheral neuropathy. *Diabetes* 62 944–952. 10.2337/db12-0716 23364451 PMC3581201

[B30] MacKnight C. Rockwood K. Awalt E. McDowell I. (2002). Diabetes mellitus and the risk of dementia, Alzheimer’s disease and vascular cognitive impairment in the Canadian Study of Health and Aging. *Demen. Geriatr. Cogn. Disord.* 14 77–83. 10.1159/000064928 12145454

[B31] Nagarajan N. Oka S. Sadoshima J. (2016). Modulation of signaling mechanisms in the heart by thioredoxin 1. *Free Radic. Biol. Med.* 109 125–131. 10.1016/j.freeradbiomed.2016.12.020 27993729 PMC5462876

[B32] Nakamura H. (2004). Thioredoxin as a key molecule in redox signaling. *Antioxid Redox Signal.* 6 15–17. 10.1089/152308604771978309 14713332

[B33] Periyasamy P. Shinohara T. (2017). Age-related cataracts: role of unfolded protein response, ca2+ mobilization, epigenetic DNA modifications, and loss of Nrf2/Keap1 dependent cytoprotection. *Prog. Retin. Eye Res.* 60 1–19. 10.1016/j.preteyeres.2017.08.003 28864287 PMC5600869

[B34] Roshan V. D. Hosseinzadeh S. Mahjoub S. Hosseinzadeh M. Myers J. (2013). Endurance exercise training and diferuloyl methane supplement: changes in neurotrophic factor and oxidative stress induced by lead in rat brain. *Biol. Sport* 30 41–46. 10.5604/20831862.1029820 24744464 PMC3944559

[B35] Saitoh M. Nishitoh H. Fujii M. Takeda K. Tobiume K. Sawada Y. (1998). Mammalian thioredoxin is a direct inhibitor of apoptosis signal-regulating kinase (ASK) 1. *EMBO J.* 17 2596–2606. 10.1093/emboj/17.9.2596 9564042 PMC1170601

[B36] Sankrityayan H. Oza M. J. Kulkarni Y. A. Mulay S. R. Gaikwad A. B. (2019). ER stress response mediates diabetic microvascular complications. *Drug Discovery Today* 24 2247–2257. 10.1016/j.drudis.2019.08.003 31430543

[B37] Schulze P. C. Yoshioka J. Takahashi T. He Z. King G. L. Lee R. T. (2004). Hyperglycemia promotes oxidative stress through inhibition of thioredoxin function by thioredoxin-interacting protein. *J. Biol. Chem.* 279 30369–30374. 10.1074/jbc.m400549200 15128745

[B38] Selkoe D. J. (2001). Alzheimer’s disease: genes, proteins, and therapy. *Physiol. Rev.* 81 741–766. 11274343 10.1152/physrev.2001.81.2.741

[B39] Sima A. A. F. (2010). Encephalopathies: the emerging diabetic complications. *Acta Diabetol.* 47 279–293. 10.1007/s00592-010-0218-0 20798963

[B40] Stamer K. Vogel R. Thies E. Mandelkow E. Mandelkow E. M. (2002). Tau blocks traffic of organelles, neurofilaments, and APP vesicles in neurons and enhances oxidative stress. *J. Cell Biol.* 156 1051–1063. 10.1083/jcb.200108057 11901170 PMC2173473

[B41] Stranahan M. A. (2015). Models and mechanisms for hippocampal dysfunction in obesity and diabetes. *Neuroence* 309 125–139. 10.1016/j.neuroscience.2015.04.045 25934036 PMC4624614

[B42] Szegezdi E. Logue S. E. Gorman A. M. Samali A. (2006). Mediators of endoplasmic reticulum stress-induced apoptosis. *EMBO Rep.* 7 880–885. 10.1038/sj.embor.7400779 16953201 PMC1559676

[B43] Tang L. Ren X. Han Y. Chen L. Meng X. Zhang C. (2020). Sulforaphane attenuates Apoptosis of hippocampal neurons induced by high glucose via regulating endoplasmic reticulum. *Neurochem. Int.* 136:104728. 10.1016/j.neuint.2020.104728 32199985

[B44] Thimmulappa R. K. Mai K. H. Srisuma S. Kensler T. W. Yamamoto M. Biswal S. (2002). Identification of Nrf2-regulated genes induced by the chemopreventive agent sulforaphane by oligonucleotide microarray. *Cancer Res.* 62 5196–5203. 12234984

[B45] Thummayot S. Tocharus C. Suksamrarn A. Tocharus J. (2016). Neuroprotective effects of cyanidin against Aβ-induced oxidative and ER stress in SK-N-SH cells. *Neurochem. Int.* 101 15–21. 10.1016/j.neuint.2016.09.016 27697517

[B46] Wang C. Lü G. Li Y. Zhao S. Huang L. (2018). Impairment of spatial learning and memory and changes of oxidative stress in hippocampus from Type 1 diabetic mice. *Zhong Nan Da Xue Xue Bao Yi Xue Ban* 43 469–474. 29886460 10.11817/j.issn.1672-7347.2018.05.002

[B47] Wang M. Zhao X. R. Wang P. Dai Y. Huang H. Zhu H. F. (2007). Glucose regulated proteins 78 protects insulinoma cells (NIT-1) from death induced by streptozotocin, cytokines or cytotoxic T lymphocytes. *Int. J. Biochem. Cell Biol.* 39 2076–2082. 10.1016/j.biocel.2007.05.022 17689130

[B48] Wang Z. Huang Y. Cheng Y. Tan Y. Wu F. Wu J. (2016). Endoplasmic reticulum stress-induced neuronal inflammatory response and apoptosis likely plays a key role in the development of diabetic encephalopathy. *Oncotarget* 7 78455–78472. 27793043 10.18632/oncotarget.12925 PMC5346653

[B49] Wu H. Ye M. Yang J. Ding J. (2016). Modulating endoplasmic reticulum stress to alleviate myocardial ischemia and reperfusion injury from basic research to clinical practice: a long way to go. *Int. J. Cardiol.* 223 630–631. 27565840 10.1016/j.ijcard.2016.08.266

[B50] Xu L. R. Liu X. L. Chen J. Liang Y. (2013). Protein disulfide isomerase interacts with tau protein and inhibits its fibrillization. *PLoS One* 8:e76657. 10.1371/journal.pone.0076657 24098548 PMC3788760

[B51] Yang B. Xu Y. Hu Y. Luo Y. Lu X. Tsui C. K. (2016). Madecassic acid protects against hypoxia-induced oxidative stress in retinal microvascular endothelial cells via ros-mediated endoplasmic reticulum stress. *Biomed. Pharmacother.* 84 845–852. 27728894 10.1016/j.biopha.2016.10.015

[B52] Yi S. Shi W. Wang H. Ma C. Zhang X. Wang S. (2017). Endoplasmic reticulum stress PERK-ATF4-CHOP pathway is associated with hypothalamic neuronal injury in different durations of stress in rats. *Front. Neurosci.* 11:152. 10.3389/fnins.2017.00152 28392758 PMC5364325

[B53] Zahraa M. A. Cruz G. L. Dickhout J. G. (2015). Crosstalk between the unfolded protein response and NF- κ B-mediated inflammation in the progression of chronic kidney disease. *J. Immunol. Res.* 2015 1–10. 10.1155/2015/428508 PMC4419235 25977931

[B54] Zargar M. A. Sheikh I. A. Ganie S. A. Ali R. Singh L. R. Gan S. H. (2014). molecular linkages between diabetes and Alzheimer’s disease: current scenario and future prospects. *CNS Neurol. Disord. Drug Targets* 13 290–298. 24059323 10.2174/18715273113126660135

[B55] Zeng X. S. Jia J. J. Kwon Y. Wang S. D. Bai J. (2014). The role of thioredoxin-1 in suppression of endoplasmic reticulum stress in Parkinson disease. *Free Radic. Biol. Med.* 67 10–18. 24140863 10.1016/j.freeradbiomed.2013.10.013

[B56] Zha X. Yue Y. Dong N. Xiong S. (2015). Endoplasmic reticulum stress aggravates viral myocarditis by raising inflammation through the IRE1-associated NF-κB pathway. *Can. J. Cardiol.* 31 1032–1040. 26111668 10.1016/j.cjca.2015.03.003

[B57] Zhao Y. Yan Y. Zhao Z. Li S. Yin J. (2015). The dynamic changes of endoplasmic reticulum stress pathway markers GRP78 and CHOP in the hippocampus of diabetic mice. *Brain Res. Bull.* 111 27–35. 25529350 10.1016/j.brainresbull.2014.12.006

[B58] Zhu W. Wang X. R. Du S. Q. Yan C. Q. Yang N. N. Lin L. L. (2018). Anti-oxidative and anti-apoptotic effects of acupuncture: role of thioredoxin-1 in the hippocampus of vascular dementia rats. *Neuroence* 379:S0306452218302203. 10.1016/j.neuroscience.2018.03.029 29592844

[B59] Zhu Y. F. Li X. H. Yuan Z. P. Li C. Y. Tian R. B. Jia W. (2015). Allicin improves endoplasmic reticulum stress-related cognitive deficits via PERK/Nrf2 antioxidative signaling pathway. *Eur. J. Pharmacol.* 762 239–246. 26049013 10.1016/j.ejphar.2015.06.002

[B60] Zschauer T. C. Matsushima S. Altschmied J. Shao D. Sadoshima J. Haendeler J. (2013). Interacting with thioredoxin-1–disease or no disease? *Antioxidants Redox Signal.* 18 1053–1062. 10.1089/ars.2012.4822 PMC3567779 22867430

